# False-positive I-131 Uptakes at Pulmonary Wedge-resection Site and Soft Tissue Lateral to the Femoral Heads in a Patient with Papillary Thyroid Carcinoma

**DOI:** 10.4274/mirt.galenos.2018.09821

**Published:** 2019-03-19

**Authors:** Bülent Yazıcı, Aylin Oral, Şeyma Alçiçek, İpek Tamsel, Ayşegül Akgün

**Affiliations:** 1 Ege University Faculty of Medicine, Department of Nuclear Medicine, İzmir, Turkey; 2 Ege University Faculty of Medicine, Department of Radiology, İzmir, Turkey

**Keywords:** Thyroid, cancer, false-positive, iodine, I-131

## Abstract

A hyper-metabolic pulmonary nodule was detected on 18F-FDG PET/CT in a 65-year-old woman who had been followed up for 12 years without any complaints following treatment for papillary thyroid cancer (PTC). Wedge resection was performed to the pulmonary nodule and the pathologic examination revealed PTC metastasis. On the post-therapeutic I-131 scan after radioiodine treatment, focal I-131 uptake was detected at the site of pulmonary wedge resection. At first, this finding was thought to be related to the residual lesion but diagnostic CT demonstrated only focal traction bronchiectasis at that region. In addition, a false-positive I-131 uptake was also detected at the soft tissue just lateral to the femoral heads probably due to inflammation.

## Abstract (tr)

On iki yıldır sorunsuz takip edilen tiroid papiller kanserli 65 yaşındaki kadın hastaya çekilen 18F-FDG PET/BT’de hipermetabolik bir akciğer nodülü saptandı. Nodül kama-rezeksiyon yöntemiyle çıkarıldı. Patoloji sonucunda bu nodülde tiroid papiller kanseri metastazı saptandı. Radyoaktif iyot tedavisinden sonra yapılan tüm vücut I-131 tarama sintigrafisinde akciğerde rezeksiyon alanında fokal I-131 tutulumu görüldü. Bu bulgunun öncelikle kalıntı bir lezyona bağlı olabileceği düşünüldü ancak daha sonra yapılan tanısal BT’de bu alanda sadece traksiyon bronşektazisi olduğu görüldü. Ayrıca, femur başlarının lateralinde yumuşak dokularda da enflamatuvar nedenlere bağlı olduğu düşünülen yanlış-pozitif I-131 tutulumları saptandı.

## Figures and Tables

**Figure 1:**
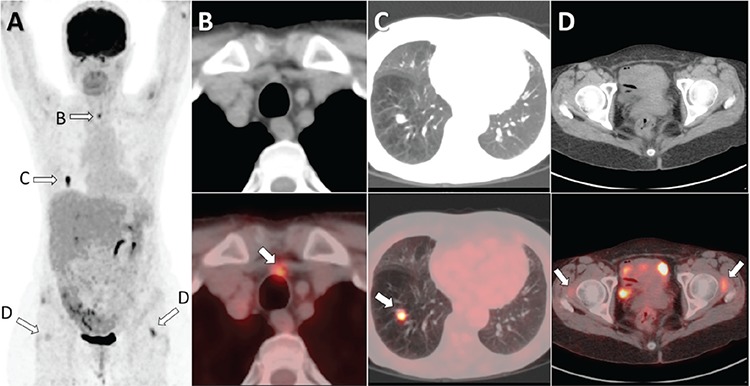
Total thyroidectomy was performed to a 65-year-old woman 12 years ago with a diagnosis of papillary thyroid cancer (PTC) with central lymph node metastasis. After the operation, 150 mCi of I-131 was given to the patient. Follow-up I-131 whole body scans (WBS) at 1-year, 3-year and 5-year were all negative. Thyroglobulin (Tg) and anti-Tg values were also negative during the WBSs. The patient had been followed for 12 years with annual ultrasound (US) without any complaints. However, a recent cervical US detected a suspicious pre-tracheal 9 mm lymph node and its biopsy revealed PTC metastasis. When thorax computed tomography (CT) was performed, a 13 mm pulmonary nodule was found in the right lower lobe. At first, since Tg was negative, the nodule was thought to be due to a lung neoplasm. Thus, ^18^F-FDG PET/CT was performed (A). PET/CT showed the hypermetabolic pre-tracheal lymph node (B) and the hypermetabolic pulmonary nodule in the right lower lobe (C). In addition, increased uptakes were detected at soft tissues lateral to the femoral heads (D) probably due to some inflammatory processes. The pulmonary nodule was completely removed by wedge-resection and pathology revealed PTC metastasis.

**Figure 2:**
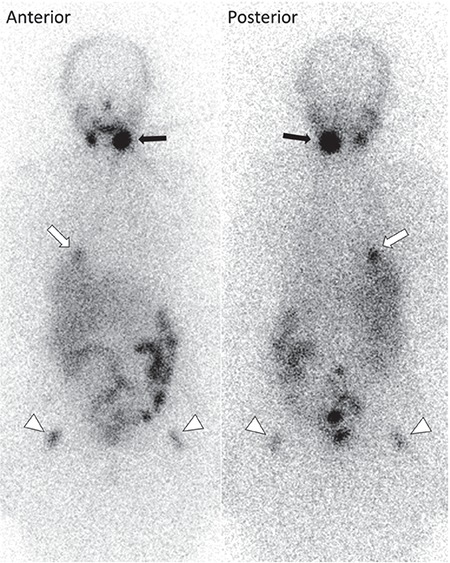
After the diagnosis of metastases, 200 mCi I-131 was applied. Tg was 77.2 ng/mL while TSH was 79 μIU/mL on the day of treatment. Post-therapeutic I-131 WBS showed focal uptakes on the right lower hemi-thorax (white-arrows) and around the hips (arrow-heads) in addition to hyperactivity on the left submandibular gland (black-arrows) and physiological gastrointestinal activities. FDG-avid metastatic pre-tracheal lymph node was false-negative on I-131 WBS.

**Figure 3:**
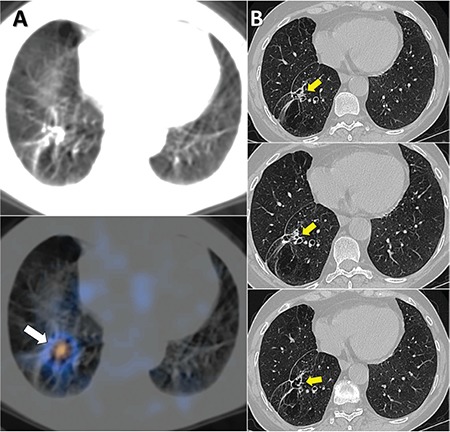
SPECT/CT images showed focal I-131 uptake at the wedge-resection site (A). A diagnostic CT was performed due to the possibility of residual lesion. Sequential slices demonstrated only focal traction bronchiectasis due to wedge-resection (B). A few case reports showed incidental detection of I-131 uptake in bronchiectasis (1,2,3). However, this case was different because we observed focal uptake at the metastasectomy site, which could be thought to be due to a residual lesion. Since the CT component of our SPECT/CT was not enough to clarify this issue, a diagnostic CT was obtained.

**Figure 4:**
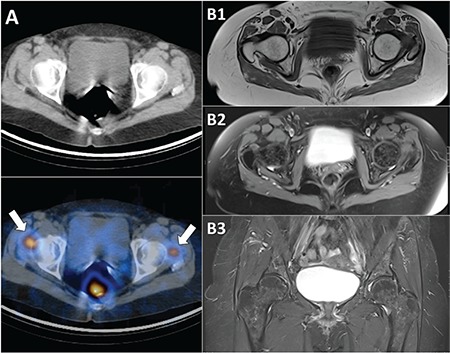
Similar to FDG-PET/CT (Figure 1 D), SPECT/CT images also showed focal uptakes on the soft tissues lateral to the femoral heads (A), which might be due to inflammation but the exact reason couldn’t be found because T1-weighted (B1) and fat-suppressed T2-weighted (B2, B3) images of magnetic resonance imaging were normal. Informed consent of the patient was obtained for all procedures. Many false-positive findings in I-131 scans due to physiological variants, inflammation or some non-thyroidal neoplasms have been reported (4,5,6,7,8). As a result, the following interesting/ rare situations were seen in combination in this case: false-positive I-131 uptakes at wedge-resection site and soft tissues, false-negative I-131 for FDGavid lymph metastasis, and detection of metastases after 12 years of disease-free follow-up that emphasizes the importance of long term follow-up. Our experience in this case also underlines the importance of careful interpretation of WBS. Focal I-131 uptake at the pulmonary wedge-resection site could be a false-positive finding due to focal traction bronchiectasis. Diagnostic CT should be performed to clarify this suspicious finding in order to determine if there is a residual lesion.
